# Genipin inhibits NLRP3 and NLRC4 inflammasome activation via autophagy suppression

**DOI:** 10.1038/srep17935

**Published:** 2015-12-11

**Authors:** Shui-Xing Yu, Chong-Tao Du, Wei Chen, Qian-Qian Lei, Ning Li, Shuai Qi, Xiao-Jing Zhang, Gui-Qiu Hu, Xu-Ming Deng, Wen-Yu Han, Yong-Jun Yang

**Affiliations:** 1 Key Laboratory of Zoonosis, Ministry of Education, College of Veterinary Medicine, Jilin University, Changchun 130062, China

## Abstract

Inflammasomes are cytoplasmic, multiprotein complexes that trigger caspase-1 activation and IL-1β maturation in response to diverse stimuli. Although inflammasomes play important roles in host defense against microbial infection, overactive inflammasomes are deleterious and lead to various autoinflammatory diseases. In the current study, we demonstrated that genipin inhibits the induction of IL-1β production and caspase-1 activation by NLRP3 and NLRC4 inflammasomes. Furthermore, genipin specifically prevented NLRP3-mediated, but not NLRC4-mediated, ASC oligomerization. Notably, genipin inhibited autophagy, leading to NLRP3 and NLRC4 inflammasome inhibition. UCP2-ROS signaling may be involved in inflammasome suppression by genipin. *In vivo*, we showed that genipin inhibited NLRP3-dependent IL-1β production and neutrophil flux in LPS- and alum-induced murine peritonitis. Additionally, genipin provided protection against flagellin-induced lung inflammation by reducing IL-1β production and neutrophil recruitment. Collectively, our results revealed a novel role in inhibition of inflammatory diseases for genipin that has been used as therapeutics for centuries in herb medicine.

Inflammasomes are cytoplasmic, multiprotein complexes that control the production of pro-inflammatory cytokines, such as IL-1β and IL-18, in response to a diverse set of inflammation-inducing stimuli, including pathogen-derived, host-derived and environmental factors^1^. Members of both the NOD-like receptor (NLR) and the absent in melanoma 2 (AIM2)-like receptor (ALR) families act as sensor molecules in inflammasomes. Activation of inflammasome sensors leads to the recruitment of ASC, an adaptor molecule, and procaspase-1; this is followed by the autoproteolytic activation of procaspase-1. Activated caspase-1 then induces cleavage of the proforms of IL-1β and IL-18 and stimulates pyroptosis^2^, a type of cell death mediated by inflammasomes.

Although inflammasomes play critical roles in anti-bacterial, anti-viral, anti-fungal and anti-parasitic immune responses, inflammasome overactivation has emerged as a critical mechanism underlying various chronic inflammatory metabolic diseases, such as atherosclerosis^3^, type 2 diabetes (T2D)^4^,^5^, gout^6^, colitis^7^, and Alzheimer’s disease^8^. Thus, regulating inflammasome activation is a promising strategy for treating inflammasome-mediated disorders; however, effective therapies to control the detrimental effects of overactive inflammasomes are lacking.

*Gardenis jasminoides* Ellis fruits have been used for centuries in traditional Chinese medicine. *Gardenis* fruits protect the liver and gallbladder and exert anti-hypertensive, anti-bleeding, and anti-swelling properties, as well as other beneficial effects. Therefore, these fruits have been extensively used to treat various diseases, including icteric hepatitis, hypertension and diabetes. Genipin, a major component in *Gardenis* fruits, also exhibits anti-inflammatory and anti-angiogenic properties^9^,^10^. Thus, genipin may regulate inflammasome activation.

Here, we demonstrated that activation of the NLR family pyrin domain-containing protein 3 (NLRP3) and CARD domain-containing protein 4 (NLRC4) inflammasomes is inhibited by genipin. Consequently, genipin is a potential therapeutic for NLRP3- and NLRC4-driven diseases.

## Results

### Genipin suppresses NLRP3 and NLRC4 inflammasome-mediated IL-1β secretion and caspase-1 activation

**Figure 1:**
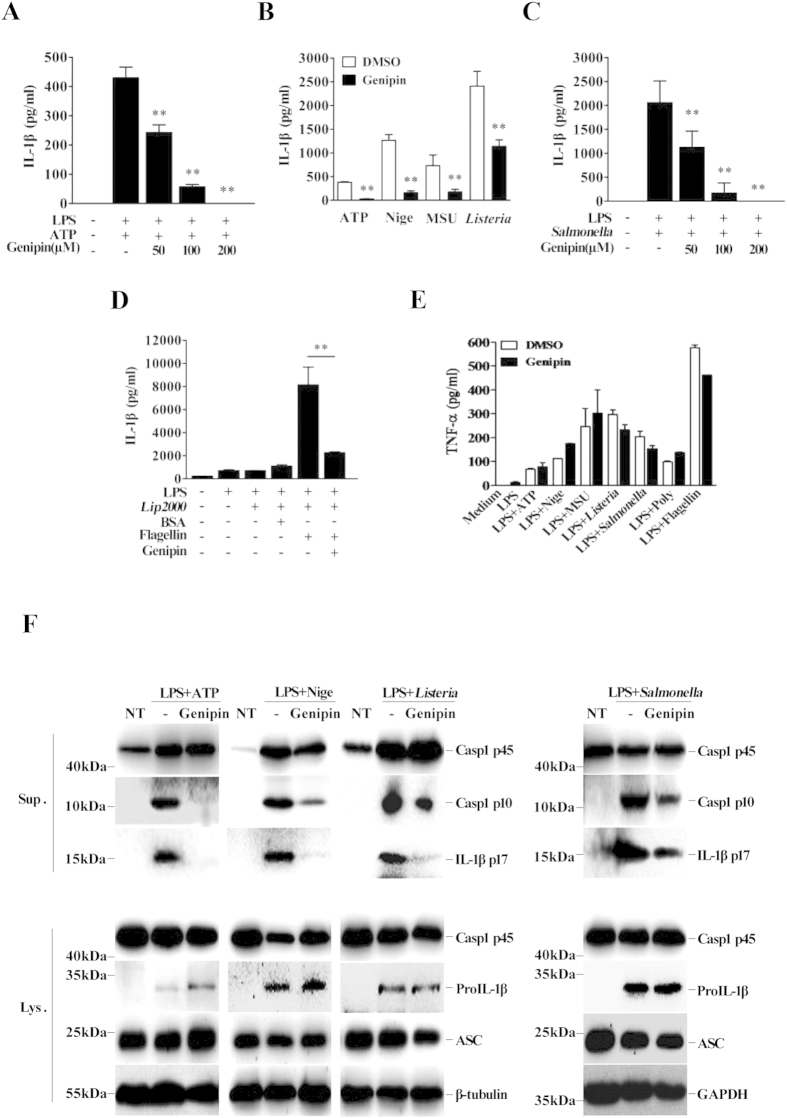
Genipin inhibits NLRP3 and NLRC4 inflammasome-mediated IL-1β secretion and caspase-1 activation in mouse macrophages. LPS-primed BMDMs were incubated with genipin (200 μM unless otherwise indicated) or DMSO for 1 h, followed by treatment with various NLRP3 or NLRC4 inflammasome agonists. Culture supernatants were analyzed for IL-1β and TNF-α by ELISA. Precipitated cell supernatants (Sup) or cell extracts (Lysate) were immunoblotted using various antibodies. (**A**) IL-1β secretion in BMDMs stimulated with the indicated doses of genipin and ATP (5 mM, 1 h). (**B**) IL-1β secretion in BMDMs stimulated with genipin plus ATP, nigericin (Nige, 20 μM, 1 h), MSU (100 μg/ml, 4 h) or *Listeria* (MOI = 20, 4 h). (**C**) IL-1β secretion in BMDMs stimulated with the indicated doses of genipin and *Salmonella* (MOI = 20, 4 h). (**D**) IL-1β secretion in BMDMs transfected with 20 μg/ml flagellin or BSA for 8 h using Lipofectamine 2000. (**E**) TNF-α secretion in BMDMs stimulated with genipin plus ATP, nigericin, MSU, *Listeria*, *Salmonella* or flagellin. (**F**) Cell supernatants and cell extracts immunoblotted for caspase-1, IL-1β and ASC. GAPDH and β-tubulin served as loading controls. The data are representative of three independent experiments. **P < 0.01.

To investigate the effect of genipin on NLRP3 inflammasome activation, lipopolysaccharide (LPS)-primed murine bone marrow-derived macrophages (BMDMs) were treated with genipin for 1 h before stimulation with ATP, a conventional NLRP3 inflammasome agonist. We found that genipin dramatically inhibited IL-1β secretion in a dose-dependent manner (Fig. 1A). In addition to ATP, a wide range of stimuli can activate the NLRP3 inflammasome. To examine whether genipin affects NLRP3 inflammasome activation via stimuli other than ATP, we treated LPS-primed BMDMs with nigericin, monosodium urate (MSU) crystals or *Listeria monocytogenes* (*Listeria*) in the presence of 200 μM genipin, a dose that did not inhibit bacterial growth or macrophage viability (Figure S1 and S3, Supplementary Information). Under these conditions, genipin also inhibited IL-1β secretion (Fig. 1B). NLRP3 inflammasome-dependent IL-1β secretion in response to nigericin or *Listeria* was confirmed using NLRP3-deficient macrophages (Figure S2A). *Listeria*-triggered IL-1β secretion was dose-dependently inhibited by genipin (Figure S2B).

To determine whether genipin-mediated IL-1β inhibition is specific to the NLRP3 inflammasome, the effects of this compound on NLRC4 inflammasome activation were assessed. *Salmonella typhimurium* (*Salmonella*) triggers the secretion of IL-1β by activating the NLRC4 inflammasome, and this effect was dose-dependently inhibited by genipin (Fig. 1C). Cytosolic bacterial flagellin is the primary trigger that activates the NLRC4 inflammasome^11^. As shown in Fig. 1D, transfection with flagellin, but not with BSA, stimulated IL-1β secretion in LPS-primed BMDMs, and genipin pretreatment significantly inhibited this induction. In contrast, LPS-dependent TNF-α secretion was not impaired by genipin (Fig. 1E).

We next evaluated IL-1β production and caspase-1 cleavage in BMDMs stimulated with ATP, nigericin, *Listeria* or *Salmonella* using immunoblotting. Consistent with ELISA results, genipin inhibited the production of mature IL-1β in cell supernatants, suggesting that genipin inhibits IL-1β processing by NLRP3 and NLRC4 (Fig. 1F). Furthermore, caspase-1 p10 levels were reduced in supernatants collected from genipin-treated BMDMs, suggesting that genipin inhibits caspase-1 activation (Fig. 1F). The dependence of IL-1β release on caspase-1 activation was confirmed using the caspase inhibitor z-VAD-fmk (Figure S2C). Collectively, the above results indicate that genipin potently inhibits NLRP3 and NLRC4 inflammasome activation. Notably, genipin treatment did not affect the expression of pro-IL-1β, pro-caspase-1 or ASC proteins in cell lysates or supernatants.

### Genipin inhibits NLRP3-mediated ASC oligomerization

**Figure 2:**
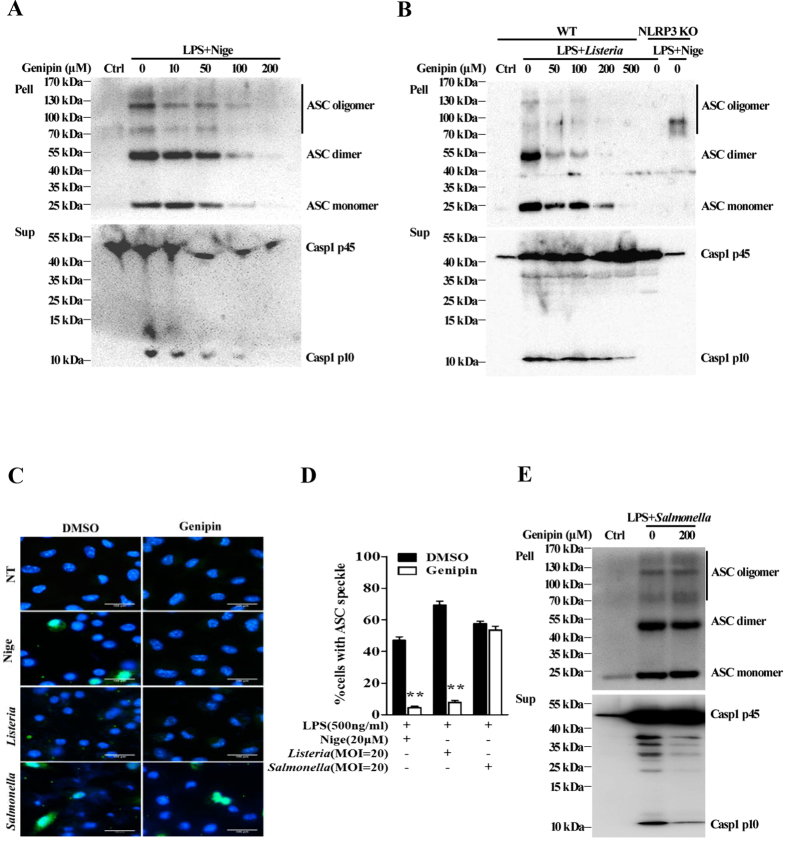
Genipin prevents NLRP3-mediated, but not NLRC4-mediated, ASC oligomerization. (**A**) LPS-primed BMDMs were pretreated with the indicated doses of genipin for 1 h and then stimulated with nigericin. The cells were extracted with 0.5% Triton X-100, and Triton-insoluble pellets were cross-linked with DSS. The cross-linked pellets were immunoblotted for ASC as described in the *Materials and Methods* section. The culture supernatants were immunoblotted for caspase-1. (**B**) LPS-primed wild-type (WT) or NLRP3 KO BMDMs were pretreated with the indicated doses of genipin, followed by *Listeria* or nigericin challenge. The cell pellets cross-linked with DSS were immunoblotted for ASC, and cell supernatants were immunoblotted for caspase-1. (**C**) LPS-primed BMDMs were stimulated with nigericin, *Listeria* or *Salmonella* in the absence or presence of genipin. The cells were fixed, permeabilized and stained for ASC (green). DAPI was used to label nuclei (blue). (**D**) The percentage of cells containing ASC speckles was quantified from three different view fields. (**E**) LPS-primed BMDMs were either not treated or pretreated with genipin, followed by *Salmonella* challenge. The cross-linked pellets were immunoblotted for ASC, and the culture supernatants were immunoblotted for caspase-1. The data are representative of three independent experiments. **P < 0.01.

We next examined ASC oligomerization, a common event associated with inflammasome activation^12^. The ASC pyroptosome, a complex of monomers, dimers and oligomers, can be cross-linked by applying disuccinimidyl suberate (DSS) to cytosolic fractions of cell lysates. Immunoblotting can then be used to detect this complex, the presence of which indicates ASC oligomerization. Alternatively, ASC oligomerization can be confirmed by the formation of ASC speckles via microscopy. In agreement with previous reports, stimulation with either LPS plus nigericin or *Listeria* induced the formation of ASC monomers, dimers and oligomers in the absence of genipin, whereas NLRP3 deficiency blocked nigericin-induced ASC pyroptosome formation (Fig. 2A,B). However, genipin dose-dependently inhibited the induction of ASC pyroptosome formation by nigericin or *Listeria*. Correspondingly, the quantity of active caspase-1 in cell supernatants was reduced by genipin. Additionally, immunostaining for endogenous ASC in macrophages showed that the induction of ASC speckles following treatment with either LPS plus nigericin or *Listeria* diminished in the presence of genipin (Fig. 2C,D). Thus, these data indicate that genipin inhibits NLRP3-dependent ASC oligomerization in addition to IL-1β secretion and caspase-1 activation.

Next, we examined the impact of genipin on NLRC4-mediated ASC oligomerization. As shown in Fig. 2C–E, stimulation with *Salmonella* induced the formation of ASC speckles and pyroptosomes in BMDMs. In contrast to its inhibitory effect on NLRP3-mediated ASC oligomerization, genipin did not affect NLRC4-mediated formation of ASC speckles or pyroptosomes, although NLRC4-mediated caspase-1 activation significantly diminished. These data indicate that the inhibitory effect of genipin on NLRC4 inflammasome activation is independent of ASC.

### Genipin inhibits autophagy-mediated inflammasome activation

**Figure 3:**
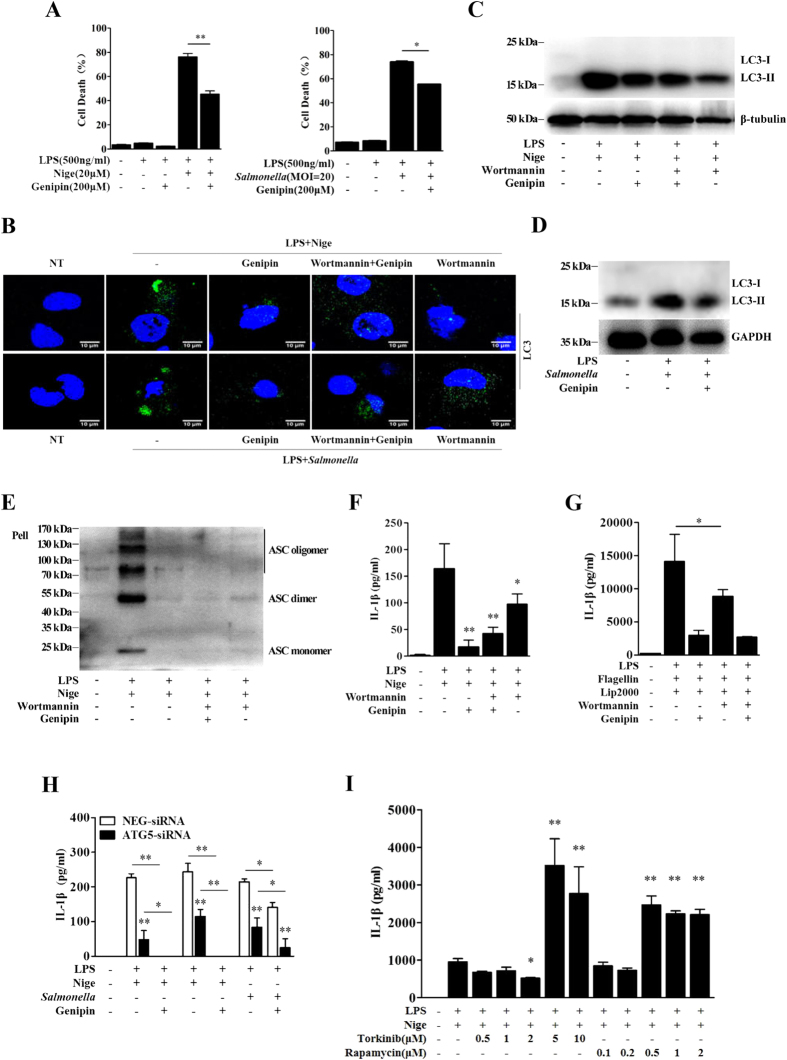
Genipin inhibits autophagy-dependent inflammasome activation. (**A**) Analysis of the cell death phenotype by Annexin-V/PI staining. LPS-primed BMDMs were pretreated with genipin and then stimulated with nigericin or *Salmonella*. The cells were stained with Annexin-V and PI. The percentage of cells positive for both Annexin V and PI is shown. (**B**) LPS-primed BMDMs were incubated with genipin and/or 5 μM wortmannin for 1 h and then stimulated with nigericin or *Salmonella*. The cells were fixed, permeabilized and stained for LC3. LC3 is shown in green, and cell nuclei are shown in blue (DAPI). (**C**,**D**) LPS-primed BMDMs were incubated with genipin and/or wortmannin and then stimulated with nigericin or *Salmonella*. The cell lysates were immunoblotted for LC3, β-tubulin or GAPDH. (**E**) LPS-primed BMDMs were incubated with genipin and/or wortmannin and then stimulated with nigericin. The cross-linked pellets were immunoblotted for ASC. (**F,G**) LPS-primed BMDMs were incubated with genipin and/or wortmannin and then stimulated with nigericin or flagellin. IL-1β secretion was measured by ELISA. (**H**) BMDMs were transfected with either siRNA targeting ATG5 or scrambled siRNA (NEG). Forty-eight hours after transfection, the cells were primed with LPS for 4 h and then stimulated with genipin before nigericin or *Salmonella* challenge. IL-1β secretion was measured by ELISA. (**I**) LPS-primed BMDMs were incubated with the indicated doses of torkinib or rapamycin for 1 h and then stimulated with nigericin. IL-1β secretion was measured by ELISA. The data are from three independent experiments conducted in triplicate. *P < 0.05 and **P < 0.01 compared with controls.

Inflammasome activation is often accompanied by caspase-1-mediated rapid cell death, which is known as pyroptosis. We examined the effect of genipin on cell death phenotype using an Annexin V/Propidium Iodide (Annexin V/PI) assay that has previously been applied to characterize pyroptosis^13^. Our results showed that cell death induction by either LPS plus nigericin or *Salmonella* was significantly inhibited by genipin (Fig. 3A and Figure S3). Furthermore, to exclude the effect of genipin on apoptotic cell death, we used immunoblotting to detect caspase-3 activation in response to various doses of genipin (Figure S3B). One limitation of the Annexin V/PI assay used above is its lack of specificity in detecting pyroptosis, as cells undergoing either autophagy or necroptosis also lose plasma membrane integrity^14^,^15^. Furthermore, the specific inhibitory effect of genipin on NLRP3-mediated, but not NLRC4-mediated, ASC oligomerization suggests that the compound may affect upstream signaling, such as the autophagy signaling pathway.

To assess autophagy, LC3 puncta formation was assessed by detecting LC3-II, a key marker of autophagy, by immunofluorescence in GFP-LC3 plasmid-transfected cells or cell lysates. Both LPS plus nigericin and *Salmonella* induced LC3 puncta formation and increased LC3-II protein level (Fig. 3B–D). In addition to inflammasome suppression, genipin also prevented autophagosome induction by nigericin and *Salmonella* (Fig. 3B–D). Wortmannin, an inhibitor of phosphatidylinositol 3-kinase, which is involved in initial autophagosome formation, was used to inhibit autophagy. Our results showed that wortmannin indeed inhibited nigericin-induced LC3 puncta formation and LC3-II protein expression (Fig. 3B,C). Similar to the effects of genipin, the inhibition of autophagy by wortmannin reduced nigericin-triggered ASC oligomer formation and IL-1β secretion (Fig. 3E,F). Wortmannin also inhibited cytosolic flagellin-induced IL-1β production (Fig. 3G). Furthermore, ATG5 knockdown by siRNA significantly prevented the production of IL-1β in response to both LPS plus nigericin and *Salmonella*. This effect was potentiated by genipin (Fig. 3H). ATG5 knockdown was confirmed by Western blotting (Figure S4A). At low doses, pretreatment of BMDMs with rapamycin, an autophagy inducer, strongly enhanced nigericin-induced IL-1β release, caspase-1 cleavage and LC3-II protein up-regulation. Conversely, at high doses, rapamycin degraded pro-IL-1β and inhibited IL-1β release. Similarly, the induction of autophagy by torkinib, an mTOR inhibitor, also promoted nigericin-mediated IL-1β production, caspase-1 cleavage and LC3-II protein up-regulation (Fig. 3I and Figure S5 A,B). Collectively, these results indicate that genipin inhibits inflammasome activation by regulating autophagy.

### Genipin inhibits UCP2 expression and promotes ROS production

**Figure 4:**
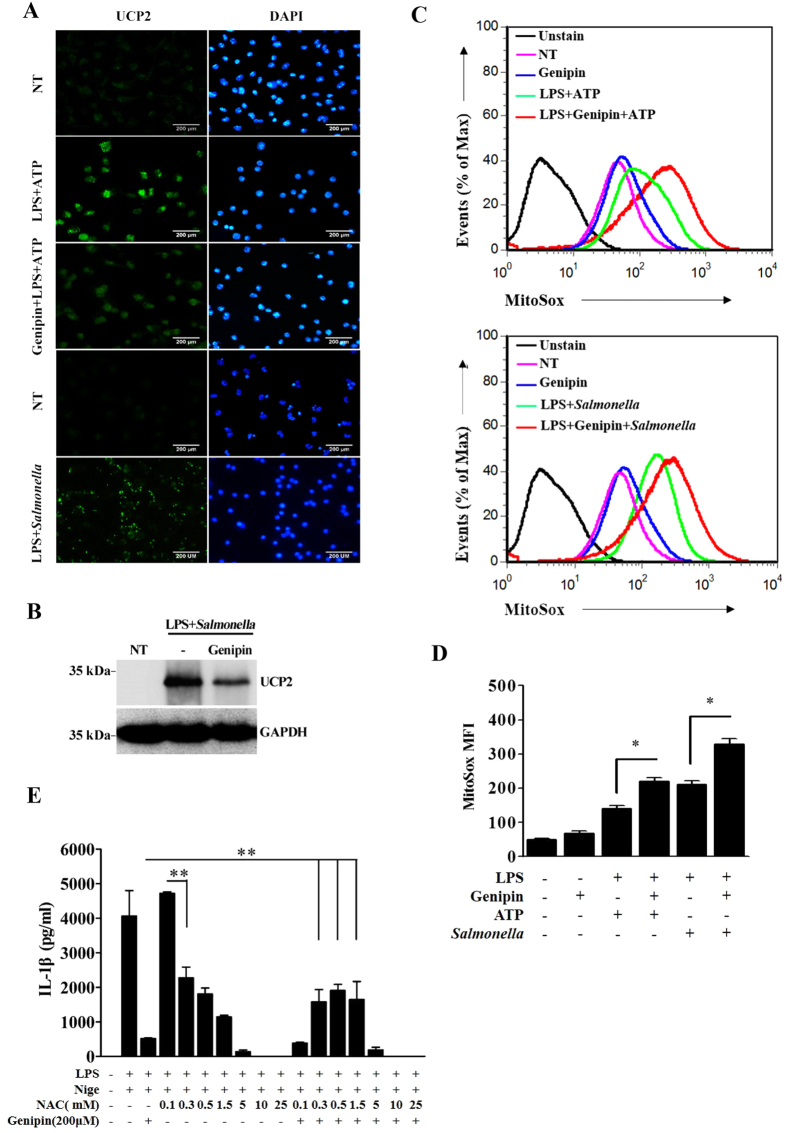
Genipin inhibits UCP2 expression and induces ROS accumulation. (**A**) LPS-primed BMDMs were pretreated with genipin and then stimulated with ATP or *Salmonella*. The cells were fixed, permeabilized and stained for UCP2 (green). DAPI was used to label nuclei (blue). (**B**) LPS-primed BMDMs stimulated with genipin and/or *Salmonella* were lysed and blotted for UCP2. (**C**) LPS-primed BMDMs were stimulated with genipin and/or ATP and/or *Salmonella*. The cells were incubated with MitoSox Red for 10 min and analyzed using a FACSAria flow cytometer. (**D**) MitoSox MFI was analyzed using FCS express. (**E**) LPS-primed BMDMs were pretreated with genipin and/or NAC at the indicated doses and then stimulated with nigericin. IL-1β release in the cell supernatants was determined by ELISA. The data are representative of three independent experiments. **P < 0.01.

Because genipin was previously categorized as an uncoupling protein 2 (UCP2) inhibitor^16^, we examined the expression of UCP2 in BMDMs stimulated with ATP or *Salmonella* in the absence or presence of genipin. By immunofluorescence and immunoblotting, we found that treatment of BMDMs with either LPS plus ATP or *Salmonella* induced UCP2 expression (Fig. 4A,B). UCP2 induction by *Salmonella* was also confirmed in human macrophage*-*like THP-1 cells and human cervical cancer cells (HeLa) (Figure S4B). Genipin treatment dramatically inhibited both ATP- and salmonella-stimulated UCP2 expression (Fig. 4A,B).

UCP2 is an inner mitochondrial membrane protein that has been shown to directly prevent the generation of reactive oxygen species (ROS)^17^. Furthermore, emerging data suggest that mitochondrial ROS play a critical role in regulating inflammasome activation^18^,^19^,^20^. Therefore, we examined mitochondrial ROS production by measuring the mean fluorescence intensity (MFI) of MitoSox Red. After 4 h of LPS priming, treatment with ATP or *Salmonella* increased mitochondrial ROS production (Fig. 4C,D). In addition, genipin enhanced ATP- and *Salmonella*-induced mitochondrial ROS production. We then used the ROS scavenger NAC to determine whether the inhibitory effects of genipin require ROS. In agreement with previous reports^21^,^22^, we observed that various doses of NAC inhibited nigericin-induced IL-1β secretion in LPS-primed macrophages (Fig. 4E). Interestingly, pretreatment with NAC at low doses reversed the inhibitory effects of genipin on nigericin-induced IL-1β secretion. These results suggested that UCP2-mediated ROS induction triggered by genipin is involved in inflammasome suppression.

### Genipin inhibits LPS- and alum-induced peritonitis and attenuates flagellin-induced lung inflammation

**Figure 5:**
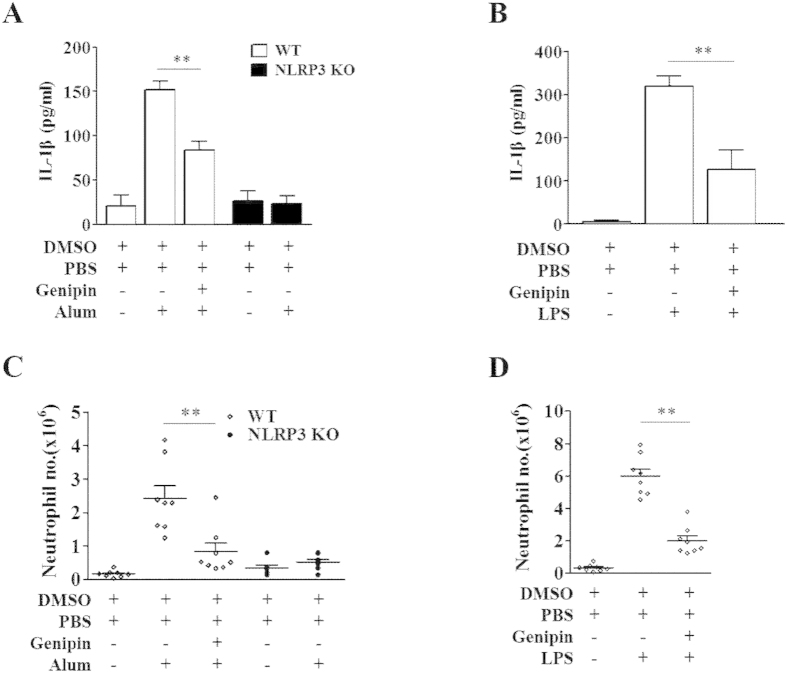
Genipin inhibits inflammatory responses to alum and LPS *in vivo*. C57BL/6J mice (n = 8 per group) and NLRP3^−/−^ mice (n = 5 per group) were i.p. injected with genipin (2 mg per body weight, i.p.) or DMSO for 1 h, followed by LPS (35 mg/kg of body weight, i.p.) or alum (2 mg per body weight, i.p.) challenge for 6 h. (**A,B**) IL-1β concentration in the supernatant of peritoneal lavage fluid was determined. (**C,D**) The number of neutrophils that had migrated into the peritoneal cavities was quantified. **P < 0.01.

We next investigated the effects of genipin *in vivo*. IL-1β induction following intraperitoneal (i.p.) injection of either LPS or alum has been previously shown to be NLRP3 dependent^23^,^24^,^25^. Thus, we examined whether genipin can block this induction of IL-1β *in vivo*. Mice were pretreated with genipin 1 h before i.p. injection of LPS or alum and were assessed 6 h later (Fig. 5A–D and Figure S6A,B). IL-1β secretion and neutrophil recruitment in the peritoneum were significantly higher in mice treated with LPS or alum than in control mice, whereas NLRP3 deficiency abolished the induction by alum. Pretreatment with genipin significantly reduced IL-1β secretion and neutrophil flux. These results indicated that genipin attenuates disease development by inhibiting NLRP3 inflammasome activation *in vivo*.

**Figure 6:**
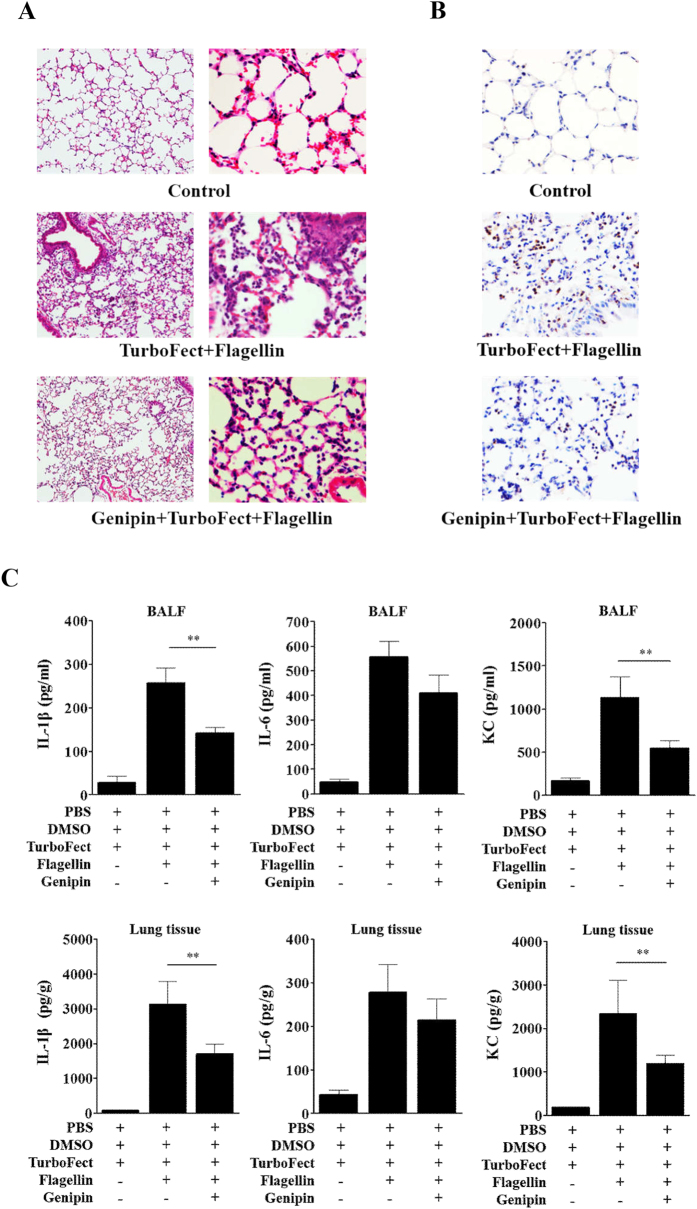
Genipin inhibits pneumonia induced by the mucosal administration of flagellin. C57BL/6J mice (n = 8 per group) were i.p. injected with genipin (2 mg per body weight, i.p.) 1 h before being intranasally instilled with a mixture of TurboFect *in vivo* transfection reagent and flagellin in a total volume of 50 μl. Lung tissue and BALF were collected 12 h after flagellin instillation. Control mice received an equivalent dose of DMSO and the mixture of PBS and TurboFect. (**A**) Lung sections were prepared and stained with H&E for histological analysis (magnification, 10/40×). (**B**) Neutrophil infiltration was detected by immunohistochemical staining for Gr-1 (magnification, 40×). (**C**) BALF and lung homogenate were collected for the analysis of IL-1β, IL-6 and KC production by ELISA. **P < 0.01.

To evaluate the role of genipin on NLRC4 inflammasome activation *in vivo*, a mouse model of flagellin-induced lung inflammation was used. Analysis of histological sections stained with H&E showed severe pneumonia in flagellin-challenged mice, and pretreatment with genipin reduced this lung pathology (Fig. 6A). Consistent with pathological changes, neutrophil accumulation in the airspaces following flagellin challenge was strikingly attenuated by genipin (Fig. 6B). We further determined the expression levels of IL-1β, IL-6 (a known downstream target of IL-1β) and KC (a neutrophil chemoattractant chemokine) in bronchoalveolar lavage fluid (BALF) and lung homogenate. We found that genipin inhibited flagellin-induced IL-1β, IL-6 and KC expression (Fig. 6C). Collectively, these data suggest that genipin reduces flagellin-mediated inflammatory responses *in vivo*. In addition, these results supported that genipin inhibits inflammasome activation in macrophages.

## Discussion

*Gardenia jasminoides* Ellis fruits have been used for centuries as an herbal medicine to relieve the symptoms of various diseases. Genipin, the major active component in *Gardenia* fruits, has been reported to exhibit anti-inflammatory activities. In the current study, we presented a new application of genipin as an anti-inflammatory agent. Our work suggests that the anti-inflammatory property of genipin may result from its ability to suppress inflammasome activation, which consequently inhibits IL-1β secretion and caspase-1 cleavage. The lack of effect of genipin on TNF-α, pro-IL-1β, pro-caspase-1 and ASC levels demonstrates that it specifically inhibits inflammasome activation.

ASC oligomerization is a common event associated with NLRP3 inflammasome activation^26^. Although NLRC4 can directly interact with pro-caspase-1 via CARD-CARD interactions, efficient pro-IL-1β processing in macrophages by the NLRC4 inflammasome still depends on ASC recruitment^27^,^28^,^29^. In the current study, genipin prevented NLRP3 agonist-induced ASC-complex formation, but it did not affect ASC-complex formation induced by stimuli that activate NLRC4.

The specific inhibition of NLRP3-mediated, but not NLRC4-mediated, ASC oligomerization suggests that genipin may affect upstream signaling, such as the autophagy signaling pathway. NLRP3 and NLRC4 inflammasome agonists significantly triggered autophagy in macrophages. Moreover, autophagy induction potentiated inflammasome-mediated IL-1β secretion and ASC oligomerization, whereas autophagy suppression by chemical or gene knockdown prevented inflammasome activation. These findings indicate that genipin inhibits inflammasome activation by suppressing autophagy in macrophages. Paradoxically, autophagy has previously been shown to both promote^30^ and inhibit NLRP3 inflammasome-mediated IL-1β secretion^31^,^32^. Our results confirmed that autophagy contributes to inflammasome activation. However, the underlying mechanism leading to this discrepancy remains elusive.

Mitochondrial ROS are critical to inflammasome activation^18^,^33^,^34^. Moreover, the UCP2 gene, a known target of genipin, encodes an inner mitochondrial membrane protein that directly prevents ROS generation^17^,^35^. Thus, we examined whether the UCP2-ROS signaling pathway is involved in the inhibitory effects of genipin. We found that agonists of NLRP3 and NLRC4 inflammasomes induced UCP2 expression and ROS production. Furthermore, genipin inhibited UCP2 expression and enhanced ROS production. The increases in ROS triggered by genipin appear to contradict its inhibitory effects on the inflammasome. We further used the ROS scavenger NAC to determine whether ROS affect the inhibitory effects of genipin on inflammasome activation. Consistent with previous reports, our results showed that NAC pretreatment inhibited nigericin-induced IL-1β secretion^18^,^33^,^34^. However, low doses of NAC reversed the inhibitory effects of genipin on nigericin-induced IL-1β secretion. In fact, there is some evidence that ROS are not absolutely required for inflammasome activation^22^. For example, the ketone metabolite β-hydroxybutyrate mediates NLRP3 inflammasome inhibition in an ROS-independent manner^36^. Furthermore, ROS induction by As_2_O_3_ is required to inhibit inflammasome activation^37^; this phenomenon is very similar to that observed with genipin. Thus, the above results indicate that UCP2-ROS signaling is involved in genipin-mediated inflammasome suppression. In addition, in THP-1 cells, genipin suppresses ATP-induced, but not nigericin-induced, NLRP3 inflammasome activation via UCP2^38^. However, our results show that genipin inhibits ATP-, nigericin-, MSU- and *Listeria*-induced NLRP3 inflammasome activation in BMDMs, as well as *Salmonella-* and flagellin-induced NLRC4 inflammasome activation. The current study did not elucidate how UCP2-ROS signaling regulates the inflammasome or autophagy; consequently, this relationship requires further investigation.

Because overactive inflammasomes play critical roles in various chronic inflammatory diseases, the identification of anti-inflammasome compounds is of clinical importance^37^,^39^. Inflammasome activation can be inhibited by several small molecules, including MCC950^25^, glyburide^40^, 3,4-methylenedioxy-β-nitrostyrene^41^, arsenic^37^ and NO^24^. We demonstrated that genipin ameliorated the severity of LPS- and alum-induced peritonitis and flagellin-induced pneumonia *in vivo*; both conditions are well-known inflammasome-mediated inflammatory diseases. Thus, genipin and other structurally related compounds may be useful anti-inflammatory agents for the treatment of inflammasome-related disorders.

In conclusion, our results demonstrated that genipin plays a prominent role in inhibiting NLRP3 and NLRC4 inflammasome activation *in vitro* and NLRP3- and NLRC4-dependent inflammation *in vivo*. Thus, genipin is a potential therapeutic for NLRP3- and NLRC4-associated syndromes and a valid reagent for further studies examining the roles of NLRP3 and NLRC4 inflammasomes in human health.

## Methods

### Mice and cells

NLRP3^−/−^ (knockout) and C57BL/6J mice were purchased from The Jackson Laboratory (Bar Harbor, ME, USA). All animal studies were conducted according to experimental practices and standards approved by the Animal Welfare and Research Ethics Committee at Jilin University. BMDMs were prepared and cultured as previously described^42^. In brief, BMDMs were isolated from the femurs of 8- to 10-week-old mice and cultured in RPMI-1640 medium containing 10% heat-inactivated FBS, 25% L929 cell-conditioned medium, 100 U/ml penicillin, and 100 U/ml streptomycin at 37 °C in a humidified atmosphere containing 5% CO_2_. The cells were harvested for assays after 7 days of differentiation.

### Bacteria

*S. typhimurium* SL1344 was a gift from Dr. Xiang-Chao Cheng (Henan University of Science and Technology, Luoyang, China). *L. monocytogenes* 10403S was a gift from Dr. Wei-Huan Fang (Microbiology Institute of Preventive Veterinary Medicine, Zhejiang University, Hangzhou, China).

### Chemicals and antibodies

Genipin, wortmannin, rapamycin, and torkinib were purchased from Selleck (Shanghai, China). Lipofectamine 2000 and Opti-MEM were purchased from Invitrogen (Carlsbad, CA, USA). LPS from *E. coli* 0111:B4, ATP, nigericin, MSU, and z-VAD-fmk were purchased from InvivoGen (San Diego, CA, USA). Alum, flagellin and N-acetylcysteine (NAC) were purchased from Sigma (Shanghai, China). Caspase-1, ASC and IL-1β antibodies were purchased from Santa Cruz Biotechnology (Santa Cruz, CA, USA). Rabbit anti-UCP2 polyclonal antibody and GAPDH were purchased from Proteintech (Wuhan, China). Rabbit anti-LC3 polyclonal antibody was obtained from Abcam (Cambridge, MA, USA). Rabbit anti-cleaved caspase-3 polyclonal antibody was purchased from Cell Signaling (Beverly, MA). Mouse anti-β-tubulin monoclonal antibody was purchased from Sungene Biotech (Tianjin, China).

### Inflammasome activation

BMDMs were primed with LPS (500 ng/ml) for 4 h in serum-free RPMI-1640 medium and then washed twice with RPMI-1640 medium. The cells were then incubated with genipin or inhibitors for 1 h before being pulsed with ATP (5 mM, 1 h), nigericin (20 μM, 1 h), MSU (100 μg/ml, 4 h), flagellin (20 μg/ml, 8 h), *Salmonella* (MOI = 20, 4 h) or *Listeria* (MOI = 20, 4 h). Flagellin was introduced into macrophages using Lipofectamine 2000 reagent. BMDMs were treated with live *Salmonella* and *Listeria* for 1 h and then washed and incubated in RPMI-1640 medium containing a cocktail of antibiotics (200 U/ml penicillin, 200 mg/ml streptomycin and 100 μg/ml gentamicin) for 3 h. After treatment, cell supernatants and cell pellets were collected for ELISA and Western blotting.

### ASC speckle detection and UCP2 staining

ASC speckles and UCP2 were detected using an indirect immunofluorescence method as previously described^43^. After stimulation, BMDMs plated on 24-well chamber slides were fixed with 4% paraformaldehyde and permeabilized with 0.1% Triton X-100. After blocking with 5% goat serum for 1 h at room temperature, the cells were incubated with anti-ASC (1:100) and Alexa Fluor^®^ 488-conjugated anti-rabbit IgG (1:1000, Invitrogen). DAPI (1 μg/ml) was used to stain nuclei. After sequential excitation, images of the same cell were saved with cellSens Dimension software and analyzed using ImageJ software.

### ASC oligomer cross-linking

For ASC oligomer cross-linking, cells were dissolved with lysis buffer (1% NP-40, 150 mM KCl and 20 mM HEPES-KOH, pH 7.5) supplemented with protease inhibitors (0.1 mM PMSF, 1 μg/ml leupeptin, 11.5 μg/ml aprotinin and 1 mM sodium orthovanadate). The lysates were centrifuged at 6000 rpm for 15 min at 4 °C. The pellets were washed twice with PBS and then suspended in 500 μl of PBS. The pellets were then cross-linked at room temperature for 30 min by adding disuccinimidyl substrate (2 mM). The cross-linked pellets were centrifuged at 13,000 rpm for 15 min and then dissolved in SDS sample buffer.

### Flow cytometric determination of cell death

Cell death was analyzed using an Annexin V apoptosis detection kit (eBioscience) as previously described^43^. Briefly, both floating and adherent BMDMs were collected and resuspended in 100 μl of Annexin V-binding buffer. After staining with 5 μl of FITC-conjugated Annexin V for 15 min at room temperature in the dark, the cells were washed and incubated with propidium iodide (PI). All cell samples were assayed using a FACSAria flow cytometer (BD Biosciences), and the acquired data were further analyzed using FCS Express (De Novo Software).

### ROS assay

To evaluate mitochondrial superoxide production, BMDMs were incubated with MitoSox Red (M36008, Invitrogen, UK) for 10 min at 37 °C in the dark, followed by inflammasome activation treatment as previously described. The cells were then washed with HBSS and analyzed using a FACSAria flow cytometer. In addition, MitoSox MFI was analyzed using FCS Express.

### Western blotting

Cell culture supernatants were precipitated by the addition of an equal volume of methanol and 0.25 volumes of chloroform and then vortexed for 10 seconds^44^. After standing for 5 minutes at room temperature, the mixtures were centrifuged at 13,000 × *g* for 10 min. The upper phase was discarded, and 200 μl of methanol was added to the interphase. The mixture was centrifuged for 10 min at 13,000 × *g*, and the protein pellets were dried at 55 °C, resuspended and boiled for 5 min at 99 °C. The samples were separated by SDS-PAGE and immunoblotted.

### Cytokine and chemokine production

The concentrations of IL-1β, TNF-α, Il-6 and KC in cell-free supernatants, lung tissues and BALF were measured by ELISA using antibody pairs from R&D Systems according to the manufacturer’s manual. The assays were independently performed three times in triplicate.

### RNA-mediated interference

For RNA-mediated interference, 7-day-old BMDMs were transfected with 30 nM validated mouse ATG-5 esiRNA (EMU038061) or negative esiRNA (both from Sigma-Aldrich) using Lipofectamine 2000 transfection reagent. Forty-eight hours after transfection, the cells were used for experiments.

### Peritonitis

Mice were i.p. injected with genipin (2 mg per body weight, i.p.), followed by LPS (35 mg/kg of body weight, i.p.) or alum (2 mg per body weight, i.p.) challenge 1 h later. Control mice received equivalent amounts of DMSO/PBS. The mice were killed 6 h later, and their peritoneal cavities were lavaged with 5 ml of PBS. The peritoneal fluids were centrifuged at 2000 rpm for 10 min, and the supernatants were analyzed for cytokine production by ELISA. The pelleted cells were stained using the May-Grünwald-Giemsa method, and the neutrophils that had migrated into the peritoneal cavities were counted. Neutrophils were also counted using another method: the pelleted cells were stained for the neutrophil surface marker Gr-1 (anti-mouse Ly-6G/Ly-6c or anti-rat IgG2b, BioLegend), followed by staining with anti-rat IgG FITC, and signals were detected using flow cytometry. The number of neutrophils was calculated by multiplying the total number of cells by the percentage of Gr-1-positive cells^45^.

### Flagellin-induced pneumonia

To induce pneumonia, mice were intranasally instilled with a mixture of flagellin and TurboFect *in vivo* transfection reagent (Thermo Scientific, China) in a total volume of 50 μl. After 12 h, BALF was obtained by lavaging the lung with 3 × 1 ml PBS containing soybean trypsin inhibitor (100 μg/ml). Cytokines and chemokines in the BALF and homogenized lung tissues were detected by ELISA. Neutrophil infiltration was detected by immunohistochemical staining, and pathological changes in the lung were detected by H&E staining.

## Additional Information

**How to cite this article**: Yu, S.-X. *et al.* Genipin inhibits NLRP3 and NLRC4 inflammasome activation via autophagy suppression. *Sci. Rep.* **5**, 17935; doi: 10.1038/srep17935 (2015).

## Supplementary Material

Supplementary Information
