# Supplementary material for: Genipin inhibits NLRP3 and NLRC4 inflammasome activation via autophagy suppression
Source: Sci Rep. 2015 Dec 11;5:17935. doi: 10.1038/srep17935 (PMC4675967; doi:10.1038/srep17935)
Supplement: Supplementary Information [file srep17935-s1.doc]

**Genipin inhibits NLRP3 and NLRC4 inflammasome activation via autophagy suppression**

Shui-Xing Yu^#^, Chong-Tao Du^#^, Wei Chen, Qian-Qian Lei, Ning Li, Shuai Qi, Xiao-Jing Zhang, Gui-Qiu Hu, Xu-Ming Deng, Wen-Yu Han, Yong-Jun Yang^*^

**Supplemental material**

**Figure S1.**

**
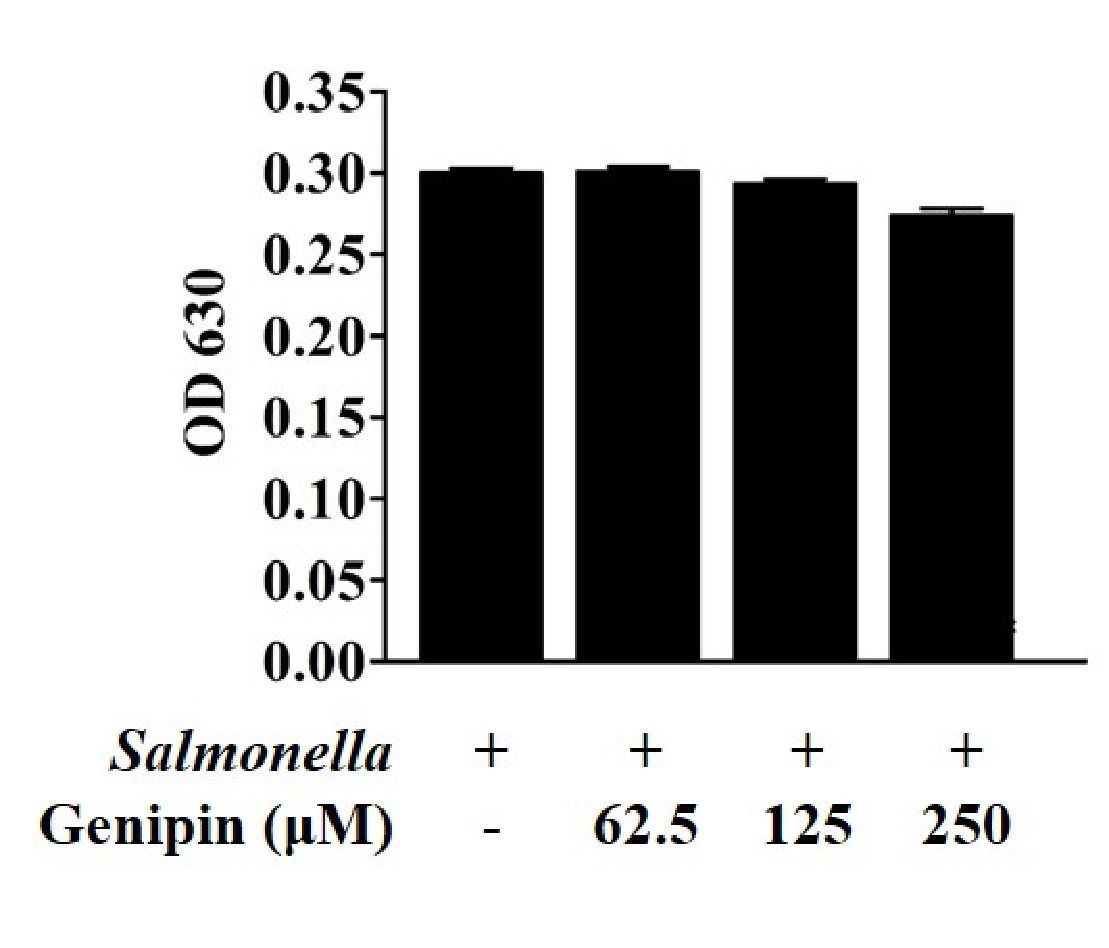

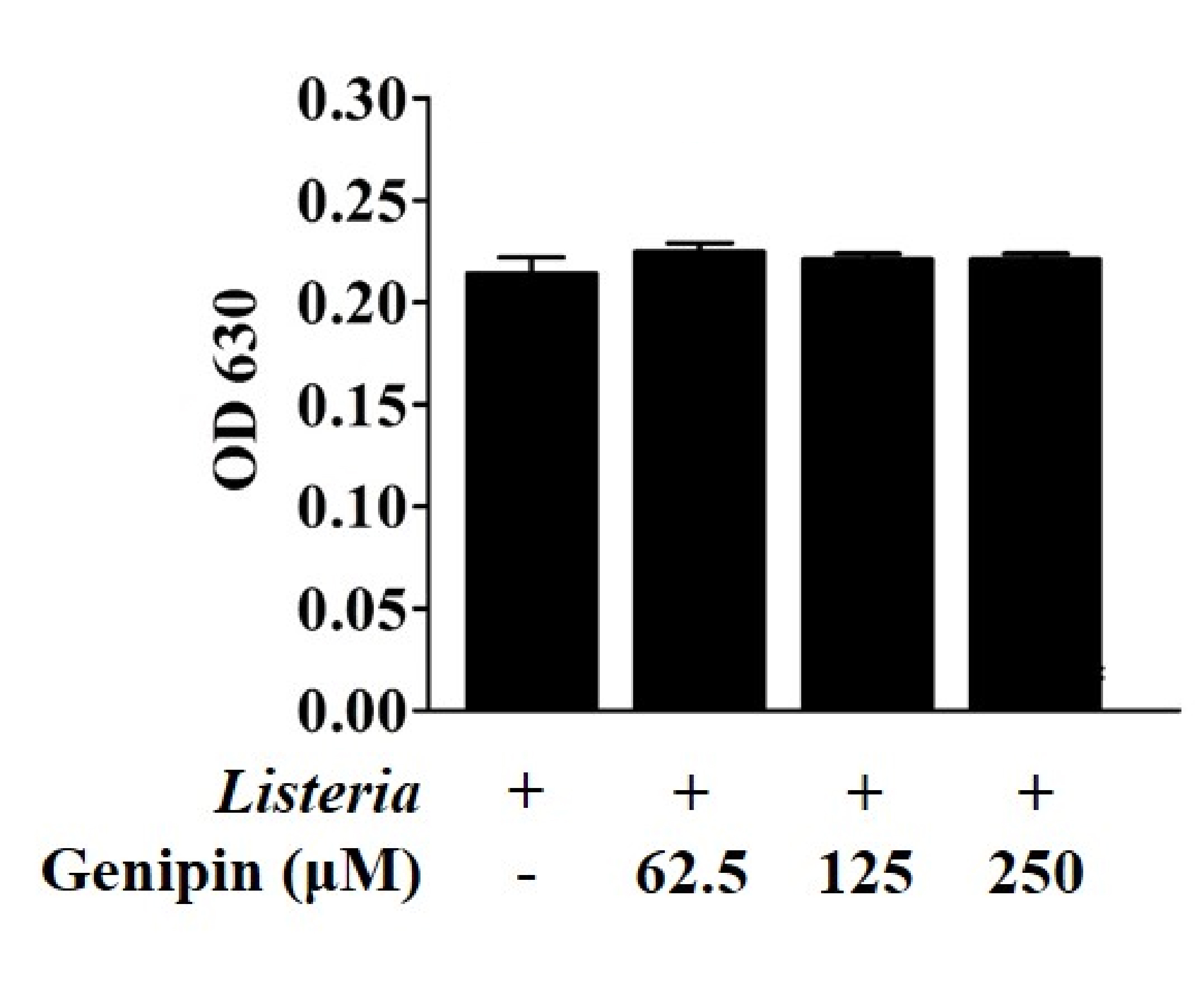
**

**Figure S1. Genipin at the selected doses was not toxic to Listeria and Salmonella growth.**To confirm that genipin primarily acted on macrophages and was not directly toxic to bacilli, *Listeria* and *Salmonella* were grown to mid-log phase and then diluted to an OD 600 of 0.1. Genipin was added at the indicated concentrations. The cultures were incubated at 37°C for 6 h, and the OD 600 was recorded. At the tested concentrations, genipin did not inhibit the growth of *Listeria* and *Salmonella.*

**Figure S2.**

**
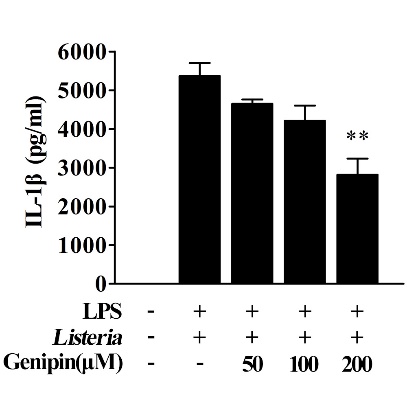

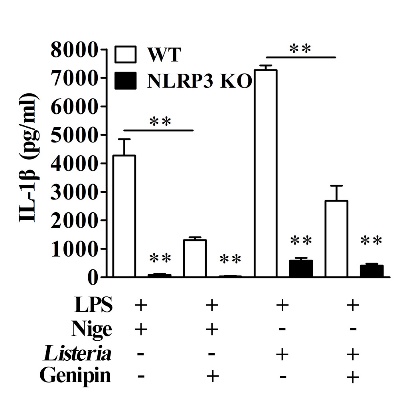
**

**A**

**B**

**
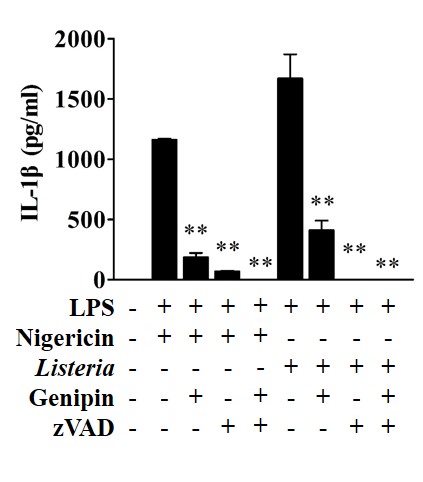
**

**C**

**Figure S2. IL-1β concentrations in cell supernatants following BMDM treatment with genipin.** (A) LPS-primed wild-type (WT) or NLRP3-deficient (KO) BMDMs were incubated with 200 μM genipin, followed by treatment with nigericin or *Listeria*. (B) LPS-primed BMDMs were incubated with various doses of genipin, followed by treatment with *Listeria*. Culture supernatants were analyzed for IL-1β by ELISA. (C) LPS-primed BMDMs were incubated with 25 μM z-VAD-fmk (zVAD) and/or genipin, followed with nigericin or *Listeria* treatment. The data are from three independent experiments conducted in triplicate. ***P* < 0.01.

**Figure S3.**

**A**

**LPS LPS+Genipin**

**FITC**

**NT LPS LPS+Genipin**

**PI**

**PI**

**LPS+Nige LPS+Genipin+Nige**

**FITC**

**LPS+*Salmonella* LPS+Genipin+*Salmonella***

**B**


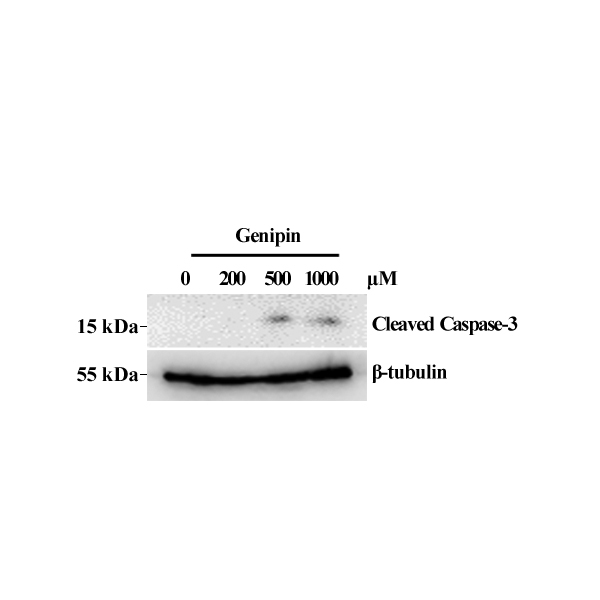


**Figure S3. Effects of genipin on LPS plus nigericin- or *Salmonella*-induced cell death in BMDMs.** (A) LPS-primed BMDMs were either untreated or pretreated with 200 µM genipin for 1 h and then stimulated with 20 μM nigericin for 1 h or infected with *Salmonella* (MOI = 20) for 4 h. The cells were stained with Annexin V and PI, washed, fixed, and analyzed by FACS. The data are presented as dual-parameter contour plots of Annexin V fluorescence (*x*-axis) versus PI (*y*-axis). Representative contour plots of three independent experiments with quadrant gates showing four populations are shown. (B) BMDMs were incubated with genipin (0 μM, 200 μM, 500 μM, or 1 mM) for 24 h. Caspase-3 activation was then detected by Western blotting. The data are from three independent experiments performed in triplicate.

**Figure S4.**

**B**

**
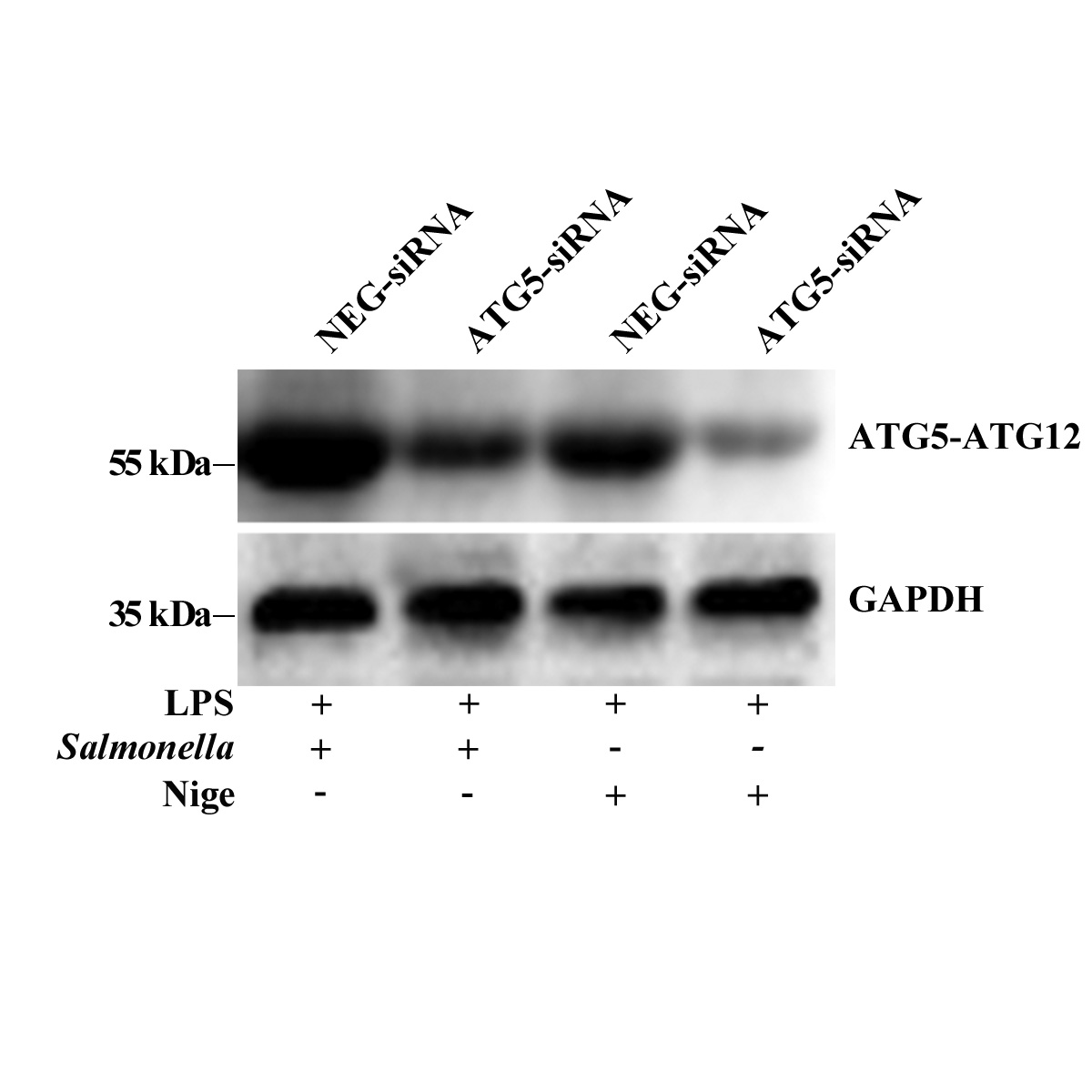

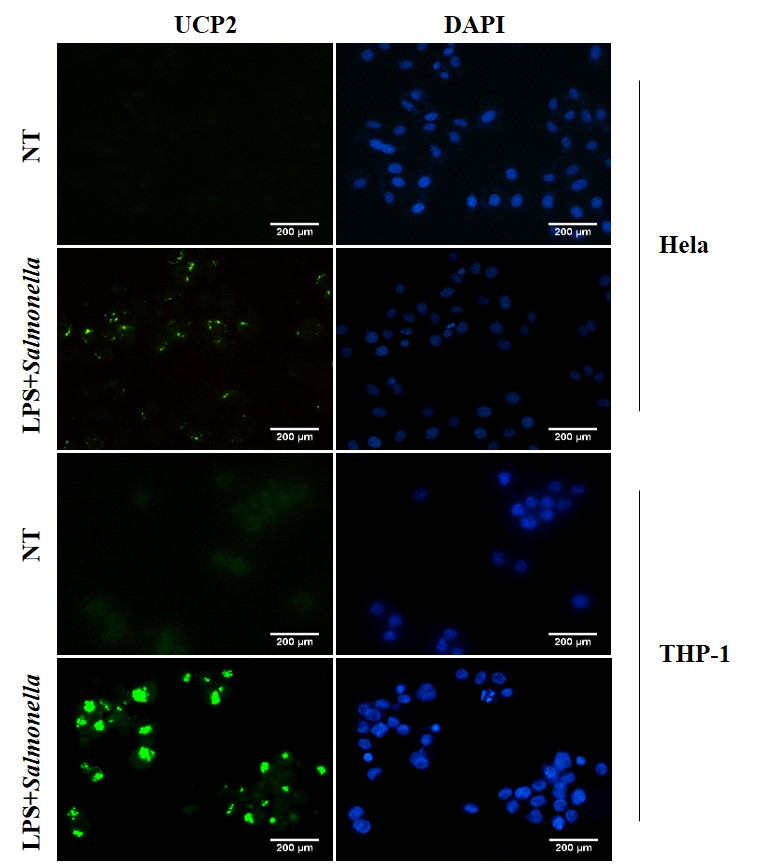
**

**A**

**Figure S4. ATG5 knockdown and UCP2 expression.** (A) siRNA-mediated ATG5 knockdown was assessed by Western blotting. BMDMs were transfected with 30 nM validated mouse ATG-5 esiRNA or negative esiRNA using Lipofectamine 2000. Forty-eight hours after transfection, the cells were treated with LPS plus nigericin or *Salmonella.* Cell lysates were prepared and analyzed for ATG5 protein expression using Western blotting. (B) Genipin inhibited UCP2 expression in HeLa and THP-1 cells. HeLa and differentiated THP-1 cells were pretreated with 200 µM genipin for 1 h and then stimulated with *Salmonella* (MOI = 20) for 4 h. The cells were fixed, permeabilized and stained for UCP2 (green). DAPI was used to label nuclei (blue). The data are representative of three independent experiments.

**Figure S5.**

**A**

**B**

**
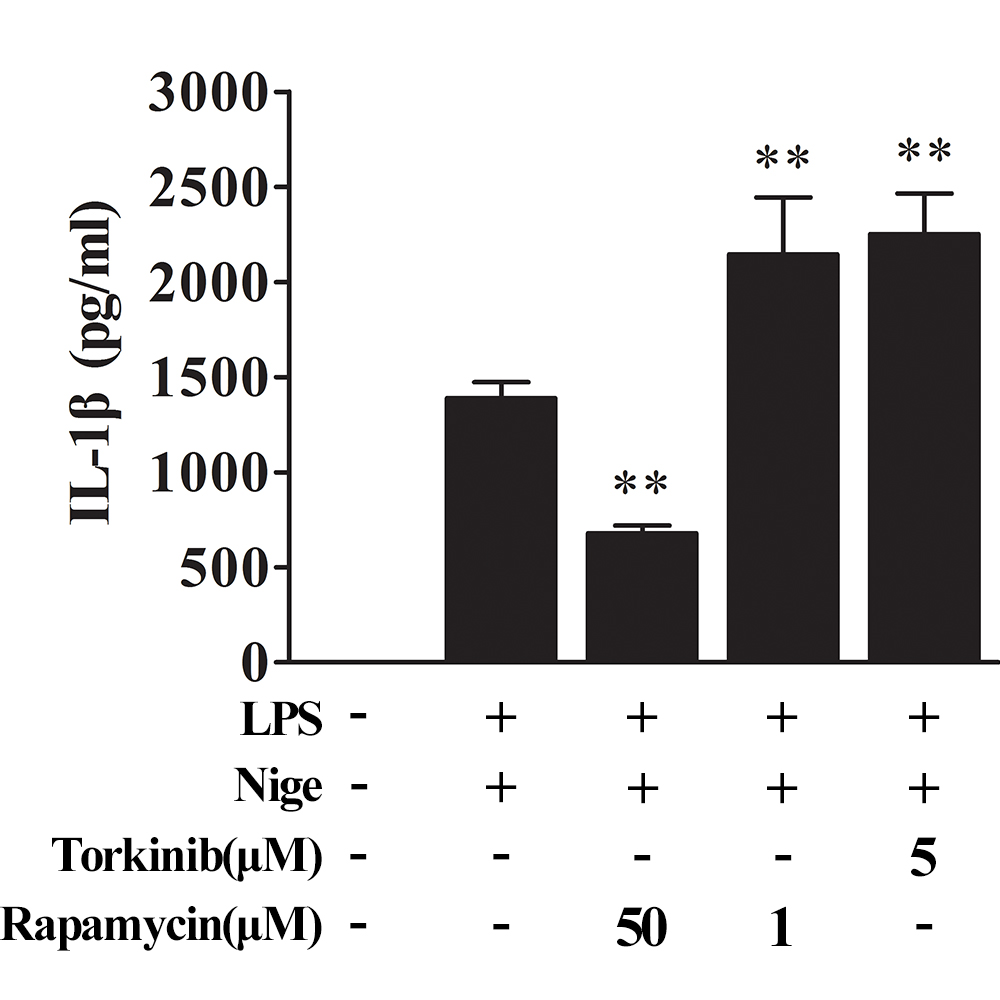
**


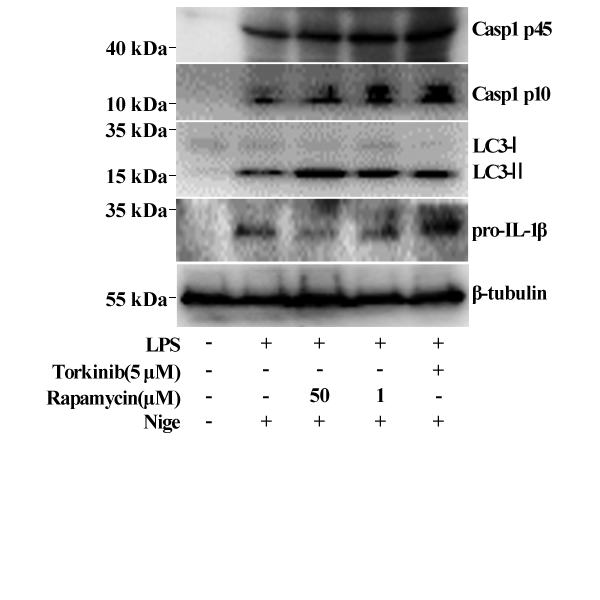


**Figure S5. Induction of autophagy promotes IL-1β** [**secretion**](javascript:void(0);) **and caspase-1 cleavage.** (A-B) LPS-primed BMDMs were either untreated or pretreated with rapamycin (1 or 50 μΜ) and torkinib (5 μΜ) for 1 h and then stimulated with 20 μM nigericin for 1 h. Culture supernatants were analyzed for IL-1β. Precipitated cell supernatants (Sup) were immunoblotted for caspase-1, and cell extracts (Lysate) were immunoblotted for LC3, IL-1β and β-tubulin. The data are from three independent experiments performed in triplicate. ***P* < 0.01.

**Figure S6.**

**A**


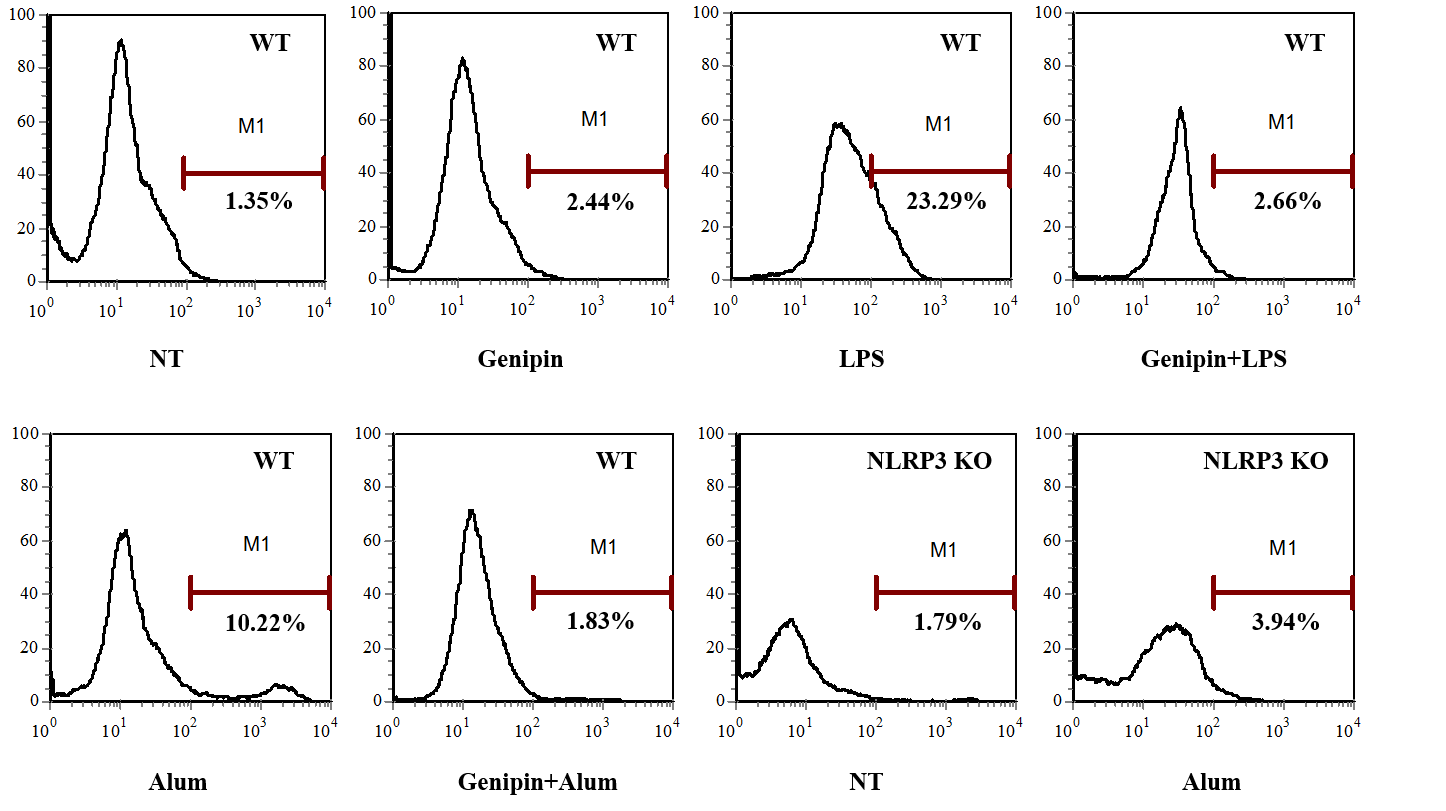


**B**


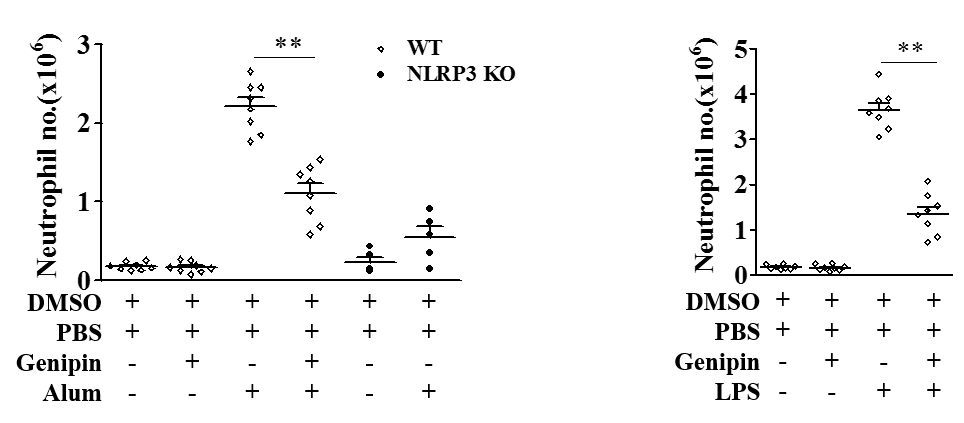


**Figure S6. Enumeration of infiltrating neutrophils using flow cytometry.** Mice were i.p. injected with genipin, followed by LPS or alum challenge 1 h later. Control mice received an equivalent dose of DMSO/PBS. The mice (WT, n=8; NLRP3 KO, n=5) were euthanized 6 h after injection, and their peritoneal cavities were lavaged. Pelleted cells were stained for the neutrophil surface marker Gr-1 (anti-mouse Ly-6G/Ly-6c or anti-rat IgG2b, BioLegend), followed by staining with anti-rat IgG FITC and detection using flow cytometry. (A) Representative flow cytometry results. (B) The number of neutrophils was calculated by multiplying the total number of cells by the percentage of Gr-1-positive cells. The data are from three independent experiments performed in triplicate. **P < 0.01.
